# Patient-specific targeted analysis of circulating tumour DNA in plasma is feasible and may be a potential biomarker in UTUC

**DOI:** 10.1007/s00345-023-04583-w

**Published:** 2023-09-18

**Authors:** Ninni Mu, Cecilia Jylhä, Tomas Axelsson, Filip Sydén, Marianne Brehmer, Emma Tham

**Affiliations:** 1 Department of Molecular Medicine and Surgery, Karolinska Institutet, Stockholm, Sweden; 2 Department of Oncology-Pathology, Karolinska Institutet, Stockholm, Sweden; 3 Clinical Genetics, Karolinska University Hospital, Stockholm, Sweden; 4 Division of Urology, Danderyd Hospital, Stockholm, Sweden; 5 Department of Urology, Stockholm South General Hospital, Stockholm, Sweden; 6 Department of Clinical Sciences, Karolinska Institutet, Stockholm, Sweden

**Keywords:** Upper urinary tract urothelial carcinoma, Prognosis, Circulating tumour DNA, Cell-free DNA, ddPCR, Targeted biomarker

## Abstract

**Purpose:**

The prognosis of upper urinary tract urothelial carcinoma (UTUC) is associated with tumour grade (G) and stage. Despite preoperative risk stratification and radical treatment, recurrence and progression are common. Thus, prognostic and monitoring biomarkers are needed. This feasibility study aimed to investigate if targeted analyses on circulating tumour DNA (ctDNA) in plasma could identify tumour-specific gene variants, and thus have potential for further evaluation as a biomarker in UTUC.

**Methods:**

Nine UTUC patients with genetically characterised tumours were included in this prospective pilot study. Two tumour-specific variants were chosen for targeted analyses with multiplex droplet digital PCR on cell-free DNA (cfDNA) from plasma at diagnosis or from recurrence.

**Results:**

Of six patients with diagnostic plasma samples, ctDNA was detected in four with G2 or G3 tumours and tumours > 300m^2^ in size. Three of these patients progressed in their disease and the fourth had the largest G3 tumour at sampling. In contrast, the two patients with undetectable ctDNA in diagnostic plasma had a G1 tumour and G3 carcinoma in situ (CIS), respectively. The patient with G3 CIS had detectable ctDNA later during follow-up and progressed thereafter with aggressive intravesical recurrence and CT-scan-verified CIS progression in the upper urinary tract. In three patients with small recurrent G1 or G2 tumours, none had detectable ctDNA in plasma and all were progression free.

**Conclusion:**

Our early findings demonstrate that ctDNA in plasma can be detected by targeted analysis in patients with UTUC. However, further studies are needed to determine its role as a potential biomarker.

**Supplementary Information:**

The online version contains supplementary material available at 10.1007/s00345-023-04583-w.

## Introduction

Although upper urinary tract urothelial carcinoma (UTUC) is rare, the incidence has been increasing during the last decades [1]. The prognosis is associated with tumour grade and stage where the 5-year survival for high-risk UTUC is < 50% and 80–90% for low-risk UTUC [2]. Radical nephroureterectomy is the standard treatment for high-risk patients whilst kidney-sparing surgery, which is coupled with lower complication risk but higher recurrence rate, can be considered for patients with low-risk tumours [2, 3]. In recent years, promising molecular biomarkers for diagnosis and risk stratification have been proposed, but none has yet been introduced in the routine clinical management of UTUC [4]. Hence, further studies on prognostic and monitoring biomarkers are warranted.

When cell death occurs by apoptosis or necrosis, small fragments of cell-free DNA (cfDNA) are released into body fluids such as plasma [5]. In cancer patients, circulating tumour DNA (ctDNA), which carries genetic alterations present in the tumour, constitutes a small proportion of the total cfDNA [5]. Liquid biopsies with analyses on ctDNA have emerged as a new promising method with a broad spectrum of potential clinical applications [5, 6]. Currently, only a handful of studies have evaluated ctDNA as a potential biomarker in UTUC [7–11]. Although the reports have highlighted the potential usefulness of ctDNA in UTUC, further research on its role as a potential biomarker is necessary. In our current pilot study, we investigated if multiplex targeted patient-specific genetic analyses on cfDNA from plasma could be a feasible approach to identify tumour-specific gene variants, and thus be a promising biomarker.

## Material and methods

### UTUC patient cohort and samples

This prospective study included nine patients diagnosed with UTUC at ureterorenoscopy (URS). Six patients underwent organ-sparing surgery; one patient (PP-6) received radiotherapy as palliative treatment, one patient received BCG-instillation in a solitary kidney with CIS (PP-7) and one patient (PP-8) underwent nephroureterectomy. Patients were diagnosed between 2018 and 2020 and were followed-up routinely (with regular URS, CT scans, urine cytology, cystoscopy and bladder wash cytology) to censor date (February 2023) or death. A next-generation sequencing (NGS) gene panel consisting of 387 genes was performed on barbotage specimens obtained at URS at diagnosis or local recurrence for genetic characterisation of all tumours prior to inclusion [12]. The NGS gene panel was performed as previously published [13]. Blood (plasma) samples were collected in conjunction with URS with verified UTUC at initial diagnosis before treatment for 6/9 patients and at the time of local recurrence for 3/9 patients. Additional plasma samples were collected for three patients at recurrence/stable disease (except for PP-1 who was tumour free when one extra plasma sample was collected). No patient had concurrent bladder cancer at the time of plasma sampling. Clinical and follow-up data were collected as described in Supplementary methods. Disease progression was defined as distant metastasis, progression to higher tumour grade or death from UTUC during follow-up and recurrence was defined as local recurrence in the upper urinary tract. The study was approved by Region Ethical Review Board in Stockholm. All patients gave their informed consent prior to inclusion.

### Extraction of cfDNA from plasma and droplet digital PCR

Extraction of cfDNA from plasma and droplet digital PCR (ddPCR) was performed as previously published and described in Supplementary methods [14]. Targets for each patient were selected based on previous tumour results from the NGS gene panel where single nucleotide variants (SNVs) or indels with highest variant allele frequencies (VAF) were primarily chosen. ddPCR mutation assays for each target labelled with fluorophores FAM for mutant allele and HEX for wildtype (wt) allele were purchased from Bio-Rad (Hercules, CA, USA) and are listed in Supplementary Table 1. Amplitude multiplex ddPCR for two targets/patient was performed on cfDNA from plasma and tumour cell DNA. Tumour cell DNA was used as positive control whilst normal cfDNA from healthy blood donors was used as negative control. ddPCR data analyses and calculations of VAF, false positive rate and concentration of mutant and wild type allele and total cfDNA (copies/mL plasma) were then performed.

## Results

### Patient characteristics

All patients had verified UTUC at the time of first plasma sample collection, 6/9 at initial diagnosis of UTUC and 3/6 at recurrence. The follow-up time after UTUC diagnosis was 4–55 months and no patient was lost to follow-up. Clinical characteristics including age at diagnosis, sex, tumour grade (G) and follow-up data of each patient are summarised in Table 1. Three patients had G1 tumours whilst two had G2 and four patients had G3 tumours. Of the patients with G2 or G3 tumours, 4/6 patients progressed in their disease during follow-up with either distant metastasis, higher tumour grade or death from disease.

**Table 1:** Patient characteristics of the nine UTUC patients included in the study

ID	Age at diagnosis	Sex (M/F)	Tumour grade WHO 1999	Tumour grade WHO 2004	Progression	Metastasis	Death from disease	Follow-up time (months)
PP-1	70	M	G1	Low	No	No	No	32
PP-2	72	M	G1	Low	No	No	No	42
PP-3	73	M	G1	Low	No	No	No	34
PP-4	84	M	G2	Low	Yes	Yes	Yes	32
PP-5	84	F	G2	High	No	No	No	44
PP-6^a^	85	M	G3	High	No	No	No	41
PP-7^b^	73	F	G3	High	Yes	No	No	38
PP-8	79	M	G3	High	Yes	Yes	No	55
PP-9	87	M	G3	High	Yes	No	Yes	4

*M* male; *F* female; *G* tumour grade

^a^Patient inoperable and received radiotherapy

^b^CIS at diagnosis and later developed intravesical recurrence and CIS progression

### Droplet digital PCR

**Fig. 1:**
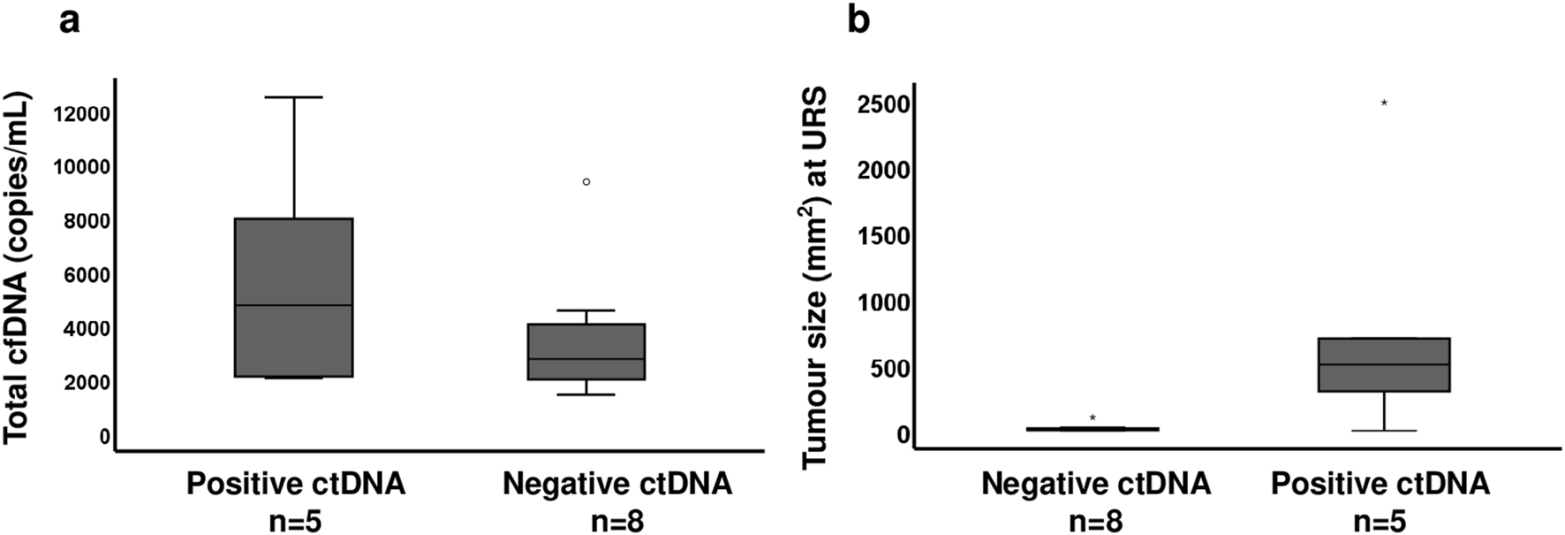
Total concentration of cfDNA and tumour size at URS at sampling in relation to ddPCR result of ctDNA in plasma. **a** Plasma samples where ctDNA was detected by ddPCR had higher median concentration of total cfDNA than samples with undetectable ctDNA. **b** Patients with detectable ctDNA in plasma had generally larger tumours at URS at sampling than the patients with undetectable ctDNA

Two SNVs or indels were selected as targets for each patient based on NGS gene panel data on barbotage tumour cells. Multiplex ddPCR assays were performed on same tumour cell DNA and also cfDNA from plasma samples (Supplementary Fig. 1). VAF, false positive rate, concentration of mutant and wild type allele and total cfDNA are listed in Table 2. In tumour cell DNA, we observed that the VAF of each genetic variant determined by ddPCR followed a similar trend as the VAF determined by NGS (Supplementary Fig. 2a). In plasma samples, the median concentration of total cfDNA was observed to be slightly higher in plasma samples where at least one of the two tumour-specific variants were detected (Fig. 1a). In addition, genetic variants detected in ctDNA from plasma were also observed to have higher median VAF in tumour cells than the variants that were not detected in plasma (Supplementary Fig. 2b).

**Table 2:** List of all assessed variants and the results obtained from ddPCR analysis of tumour cell DNA (one sample/patient) and cfDNA in plasma from each patient. Each patient had at least one plasma sample and two variants were analysed for each sample and shown in separate rows. If multiple plasma samples were available from a patient, the results are shown as additional rows

ID	Variant	Tumour cell DNA	False positive rate	Plasma cfDNA					Total cfDNA
		Mutant VAF (%)	VAF (%)	Mutant VAF (%)	Mutant Copies/mL	WT Copies/mL	Variant result	Sample result	Copies/mL
PP-1	*KMT2D* c.15517G > T	7	0	0	0	4177	Negative	Negative	9329
	*KMT2D* c.6562delG	5	0.05	0	0	5152	Negative		
	*KMT2D* c.15517G > T			0	0	1396	Negative	Negative	3105
	*KMT2D* c.6562delG			0	0	1709	Negative		
PP-2	*KDM6A* c.1663C > T	5	0	0	0	483	Negative	Negative	1422
	*TERT* C228T	4	0.07	0.04	0	939	Negative		
	*KDM6A* c.1663C > T			0	0	777	Negative	Negative	2262
	*TERT* C228T			0	0	1485	Negative		
PP-3	*KDM6A* c.3373dupA	20	0.03	0	0	753	Negative	Negative	3502
	*FGFR3* c.1118A > G	17	0	0	0	2749	Negative		
PP-5	*HRAS* c.351G > C	9	0	0	0	2105	Negative	Negative	4557
	*TERT* C228T	6	0	0	0	2452	Negative		
PP-6	*TP53* c.326 T > C	19	0	4	217	5767	Positive	Positive	12477
	*PIK3CA* c.1633G > C	7	0	1	83	6410	Positive		
PP-4	*KMT2C* c.4432C > T	22	0.02	0.03	1	2079	Negative	Positive	4744
	*TERT* C228T	20	0.02	24	645	2020	Positive		
PP-8	*TSC1* c.2507C > G	24	0	1	14	1037	Positive	Positive	2052
	*TERT* C228T	21	0.02	1	9	993	Positive		
PP-7	*KDM6A* c.3293delT	27	0	0	0	630	Negative	Negative	1740
	*TERT* C228T	14	0.05	0	0	1110	Negative		
	*KDM6A* c.3293delT			0	0	947	Negative	Negative	2418
	*TERT* C228T			0	0	1471	Negative		
	*KDM6A* c.3293delT			0.4	3	822	Positive	Positive	2103
	*TERT* C228T			0.3	4	1274	Positive		
PP-9	*ELF3* c.740G > A	19	0.02	0	0	3931	Negative	Positive	7943
	*KMT2D* c.11024delG	12	0.05	0.2	8	4004	Positive		

*VAF* variant allele frequency; *wt *wild type

### Plasma ctDNA detection

**Fig. 2:**
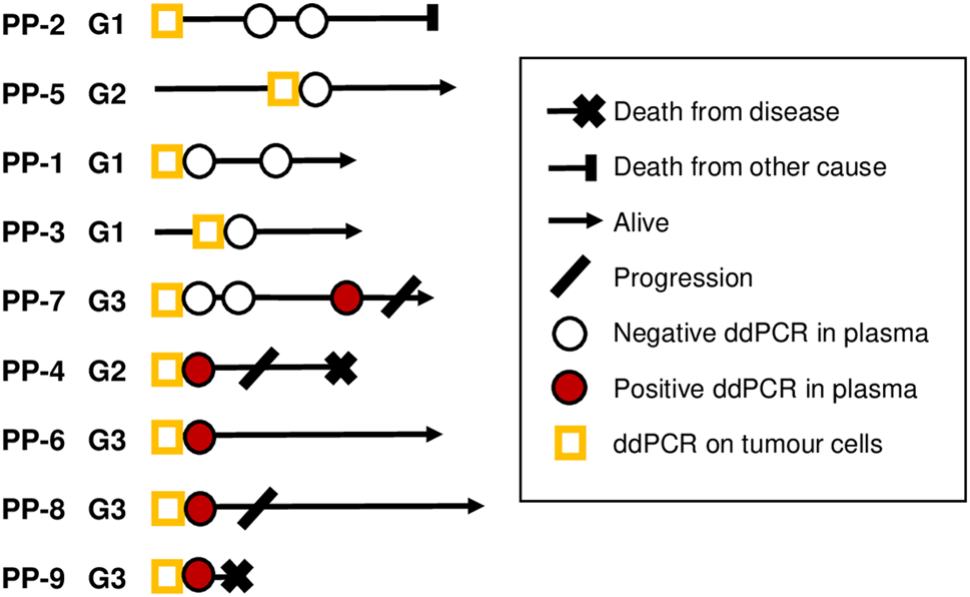
Results from ddPCR of ctDNA in plasma in relation to clinical course and outcome for all included patient with UTUC. Tumour-specific gene variants were detectable with ddPCR in plasma from five patients during their clinical course, of which four of them later progressed in their disease. In contrast, none of the patients with undetectable variants in plasma throughout the follow-up period progressed in their disease. *G* tumour grade

Tumour-specific variants in cfDNA from plasma were analysed by multiplex ddPCR in 6/9 patients at initial UTUC diagnosis and at recurrent disease for the remaining 3/9 patients. In addition, more than one plasma sample was analysed for three patients. The results from ddPCR in relation to clinical status and outcome are presented in Fig. 2 and Table 3. For the diagnostic cfDNA samples, at least one of two variants were detectable in 4/6 patients, three of whom had G3 tumours at diagnosis. Two of these three patients later progressed in their disease. The remaining patient with detectable ctDNA at diagnosis had a G2 tumour but later also progressed with metastasis and died from disease. In contrast, the two patients with undetectable variants in plasma at diagnosis had a G1 tumour and a stable but BCG-resistant G3 carcinoma in situ (CIS) in a solitary kidney. The patient with G1 tumour also had a plasma sample taken during follow-up where the patient was tumour-free and ctDNA in plasma was not detected. Interestingly, the patient with G3 CIS had detectable variants in ctDNA on a third sampling time point with stable CIS and progressed 10 months thereafter with aggressive intravesical recurrence and CT scan verified CIS progression in the upper urinary tract. The remaining three patients with initial sampling at recurrence had G1 (*n* = 2) or G2 (*n* = 1) tumours and were progression free at follow-up. None of these three patients had detectable ctDNA in plasma.

**Table 3:** Patient status and tumour size at URS at sampling in relation to ctDNA in plasma. Multiple sampling time-points from the same patient are listed as separate rows

ID	Tumour grade	Status at sampling	Tumour size (mm^2^)	Plasma ctDNA
PP-6	G3	Diagnosis	2491	Positive
PP-4	G2	Diagnosis	700	Positive
PP-8	G3	Diagnosis	500	Positive
PP-9	G3	Diagnosis	300	Positive
PP-1	G1	Diagnosis	100	Negative
		Tumour-free^b^	0	Negative
PP-5	G2	Recurrence	25	Negative
PP-3	G1	Recurrence	15	Negative
PP-2	G1	Recurrence	6	Negative
		Recurrence	12	Negative
PP-7	G3	Diagnosis	0 (CIS)	Negative
		Stable CIS	0 (CIS)	Negative
		Stable CIS	0 (CIS)^a^	Positive

*G* grade; *CIS* carcinoma in situ

^a^Progression with increased contrast loading in thickened ureteric and renal pelvic wall by CT scan

^b^No visible tumour in bladder or upper urinary tract

Tumour size was estimated by URS at the time point of each plasma sampling (Table 3). We observed that patients with detectable ctDNA in plasma had larger median tumour size at URS than patients with undetectable ctDNA (Fig. 1b). For patients with undetectable ctDNA in plasma, the largest tumour size at URS was 100 mm^2^. This specific plasma sample was taken at diagnosis in a patient with G1 tumour. In contrast, in patients where ctDNA was detected, the smallest tumour size at sampling was 300 mm^2^. The only exception was the third sampling time point that was positive for ctDNA in the patient with G3 CIS where the tumour was macroscopically undetectable. Amongst the patients with detectable ctDNA at diagnosis, the largest tumour size at sampling was 2491 mm^2^. This specific patient did not suffer disease progression, but had a large inoperable G3 tumour at the time of sampling and later received palliative treatment with radiotherapy.

## Discussion

Although a number of studies have been performed assessing the utility of ctDNA as a prognostic and monitoring biomarker in urothelial carcinoma with promising results [15–17], only a few studies have focussed on UTUC [7–11]. These have either used NGS or singleplex ddPCR assays for genetic analysis of ctDNA. In our current feasibility study, UTUC patients with genetically characterised tumours by NGS on tumour cells from barbotage specimens were included to determine whether custom-designed targeted multiplex ddPCR on ctDNA in plasma is a feasible approach to identify previously known tumour-specific gene variants.

In our pilot cohort, where all had detectable tumour-specific gene variants in tumour cells received from focal barbotages, 5/9 patients had detectable ctDNA in plasma during their clinical course. Four of them, including one patient with CIS, later progressed in their disease with either distant metastasis, progression to higher tumour grade or death from UTUC 4–12 months after the sampling time point, which might suggest that ctDNA could have potential as a biomarker for disease progression. These results are consistent with previous findings where ctDNA was found in patients with metastatic UTUC and was shown to predict UTUC patients with poor prognosis [7, 8]. The remaining patient with detectable ctDNA did not progress but had the largest tumour size at URS in the entire cohort. We also observed a trend between tumour size and ctDNA detection, as all with detectable ctDNA (except the patient with CIS) had large macroscopically visible tumours at the time of plasma sampling. It is also known that tumour burden is correlated with ctDNA levels in plasma [18]. However, if this is also applicable for UTUC remains to be further explored.

In this study, targeted multiplex ddPCR was used to detect known patient-specific gene variants in plasma. This method offers a fast, high-sensitivity, minimally invasive and easy-to-interpret approach to detect a few patient-specific variants at once and could, thus, serve as a promising method for surveillance of UTUC if deemed clinically relevant. The only recurrent mutation analysed in the cohort was the *TERT* promoter mutation C228T, which highlights the need to perform genetic testing on tumour material to select patient-specific genetic variants for monitoring tests. NGS analysis of tumour cells will likely become part of future clinical routine as the cost of sequencing comes down which will simplify the selection of tumour-specific gene targets. Tumour genetic heterogeneity and subclonal genetic evolution during progression affect the genetic landscape and level of ctDNA [17] and are factors that could potentially influence the interpretation of results by this targeted approach. To overcome this issue, we chose to analyse two variants with high VAF in tumour cells determined by NGS for each patient as we speculated that those variants were more likely to be genetic driver events and less likely to be lost during tumour evolution. In 2/5 patients with detectable ctDNA, only one variant was detected in plasma, highlighting the need to screen for several targets. Also, based on our current observations, it is possible that the total cfDNA concentration in plasma could influence the results.

## Conclusion

Detection of tumour-specific gene variants in ctDNA from plasma by targeted multiplex ddPCR is a feasible method in patients with UTUC. Our early observations suggest that detection of ctDNA by this minimally invasive approach could have potential as a biomarker in UTUC. However, no clinical conclusions can be drawn and further studies are warranted to determine its role as a biomarker.

### Supplementary Information

Below is the link to the electronic supplementary material.

Supplementary file1 (PDF 5674 KB)

Supplementary file2 (PPTX 81 KB)

Supplementary file3 (DOCX 15 KB)

Supplementary file4 (DOCX 14 KB)

## Data Availability

Not applicable.
