# Supplementary material for: Patient-specific targeted analysis of circulating tumour DNA in plasma is feasible and may be a potential biomarker in UTUC
Source: World J Urol. 2023 Sep 18;41(12):3421–7. doi: 10.1007/s00345-023-04583-w (PMC10693512; doi:10.1007/s00345-023-04583-w)

Supplementary figure 1

2D plots from amplitude multiplex ddPCR on tumour cells (positive control), normal plasma (negative control) and cfDNA from plasma for all nine included patients with UTUC. All targets were labelled with FAM for mutant alleles and HEX for wildtype (wt) alleles.

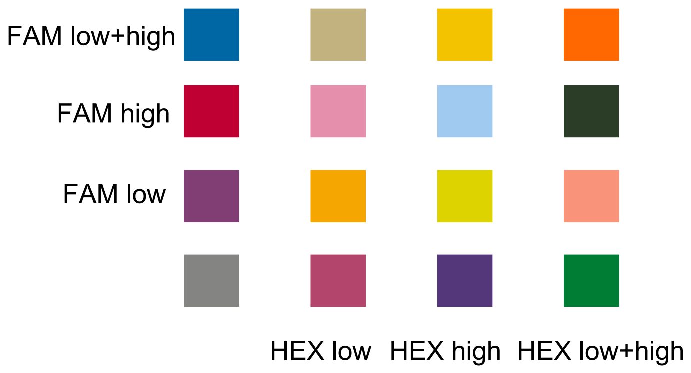

PP-1

FAM/HEX high: *KMT2D* c.15517G>T
FAM/HEX low: *KMT2D* c.6562delG

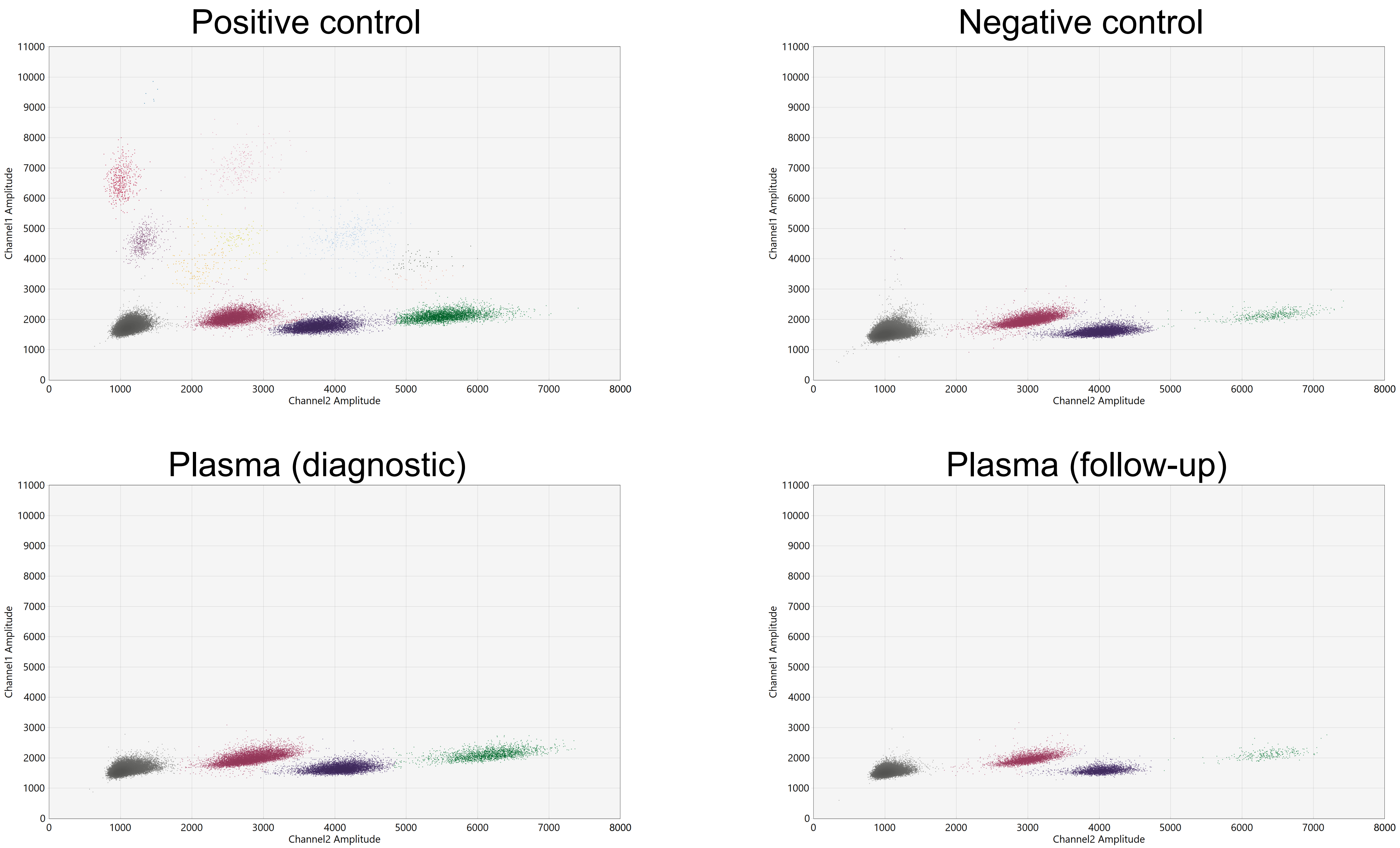

PP-2

FAM/HEX high: *KDM6A* c.1663C>T
FAM/HEX low: *TERT* C228T

Positive control

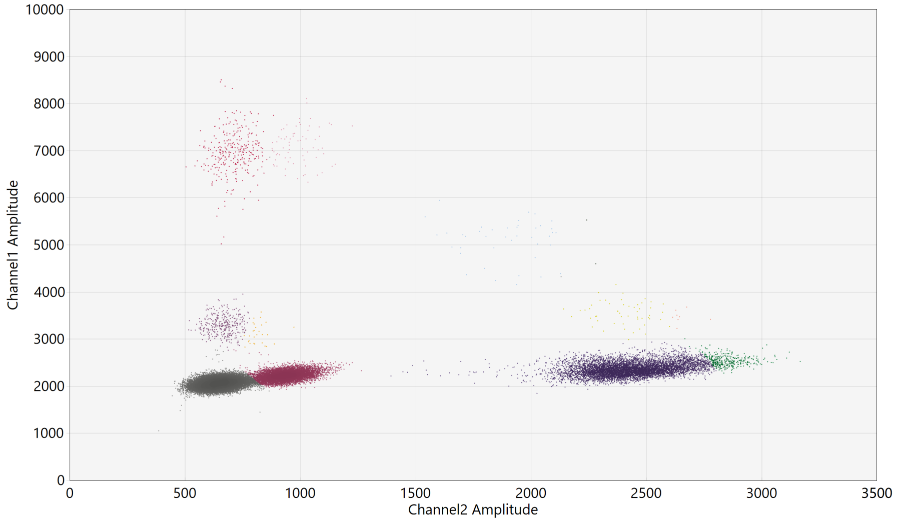

Negative control

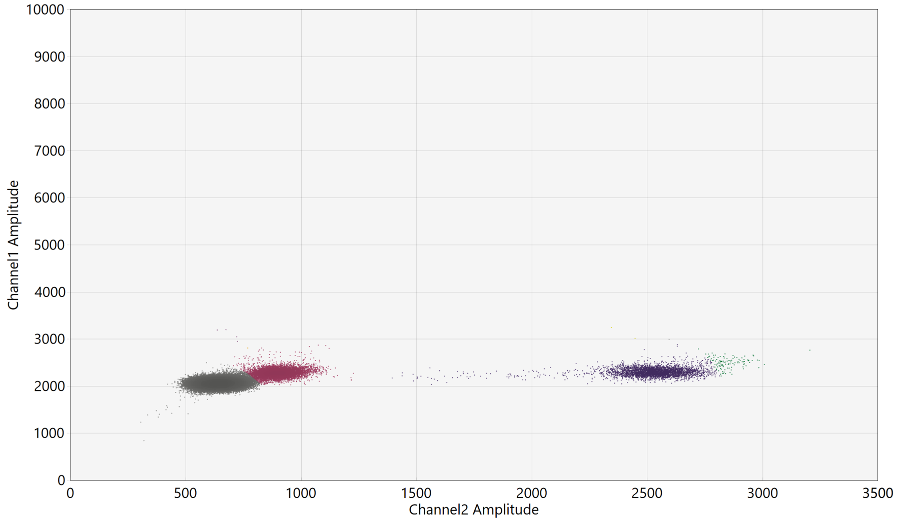

Plasma (follow-up)

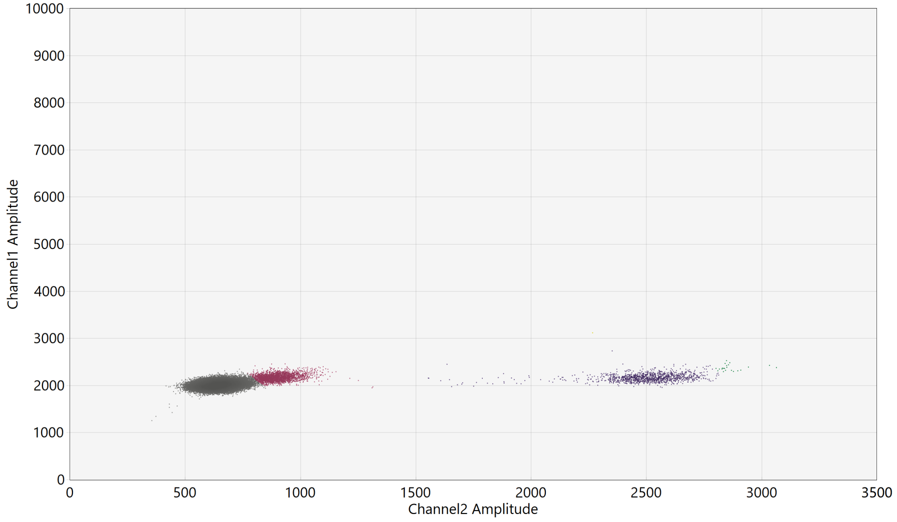

Plasma (follow-up)

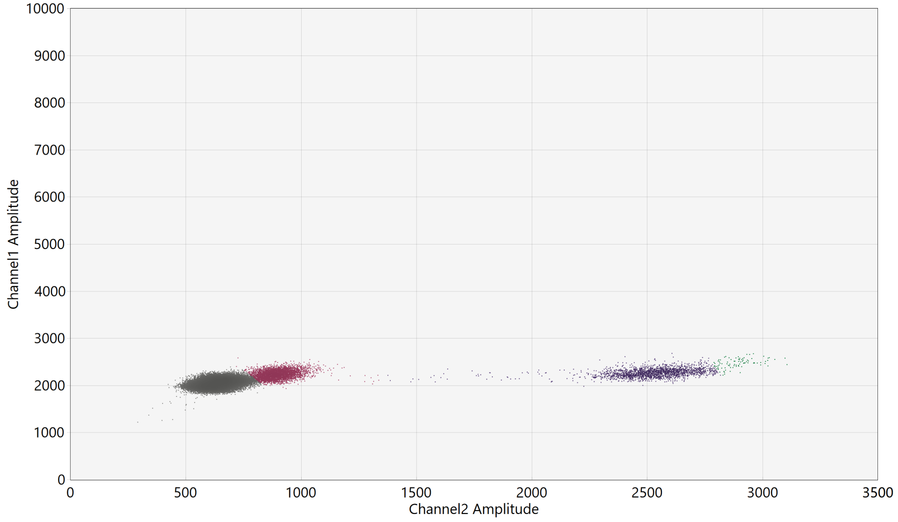

PP-3

FAM/HEX high: *KDM6A* c.3373dupA
FAM/HEX low: *FGFR3* c.1118A>G

Positive control

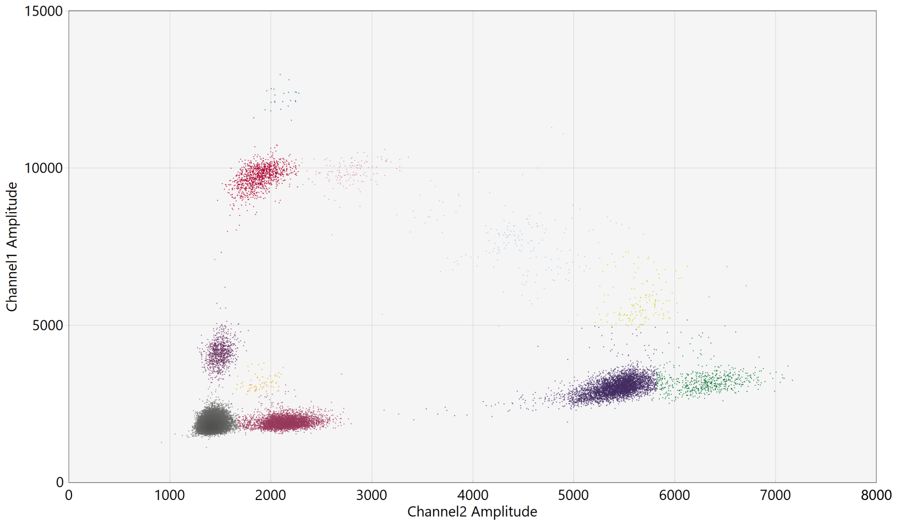

Negative control

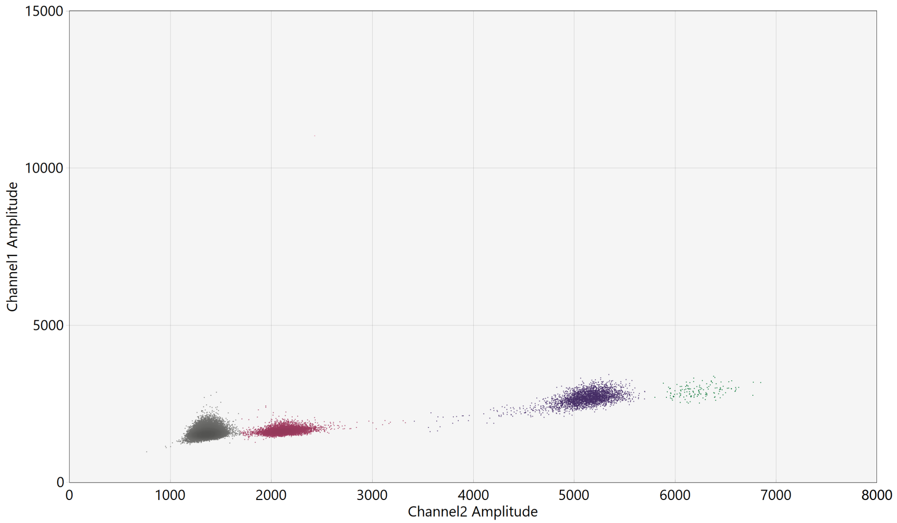

Plasma (follow-up)

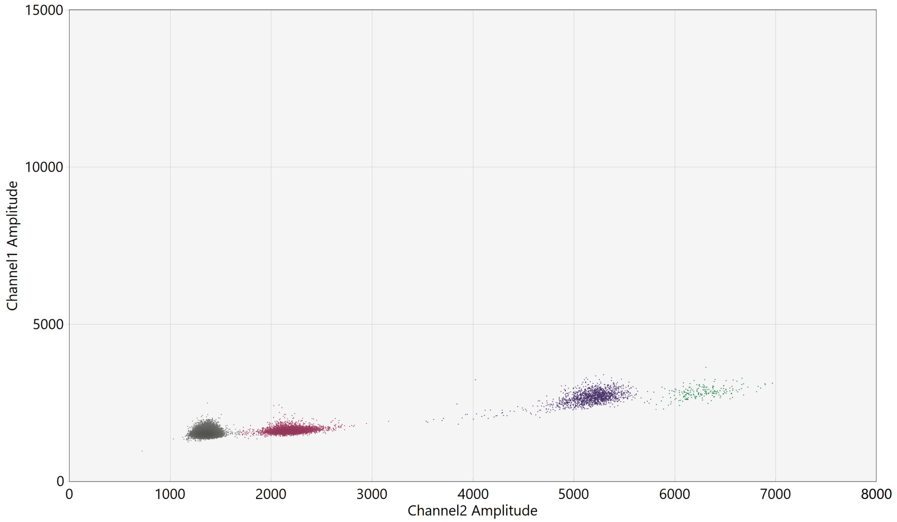

PP-4

FAM/HEX high: *KMT2C* c.4432C>T
FAM/HEX low: *TERT* C228T

Positive control

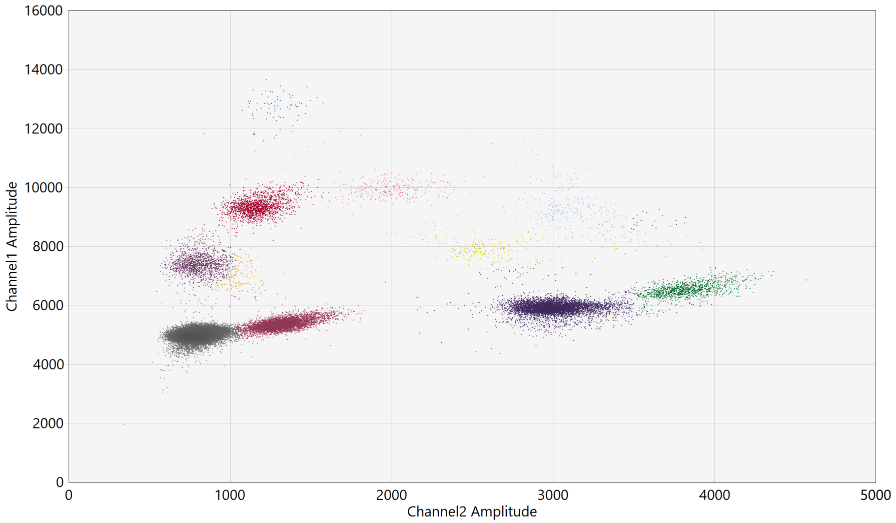

Negative control

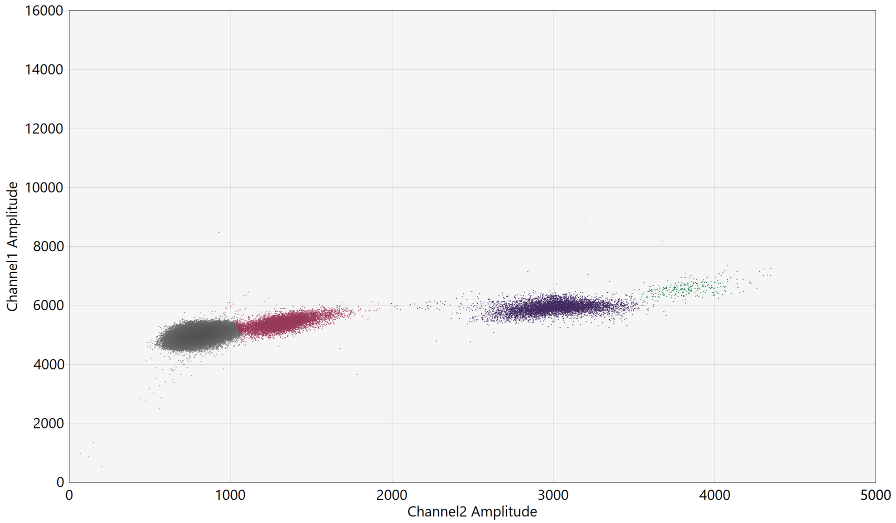

Plasma (diagnostic)

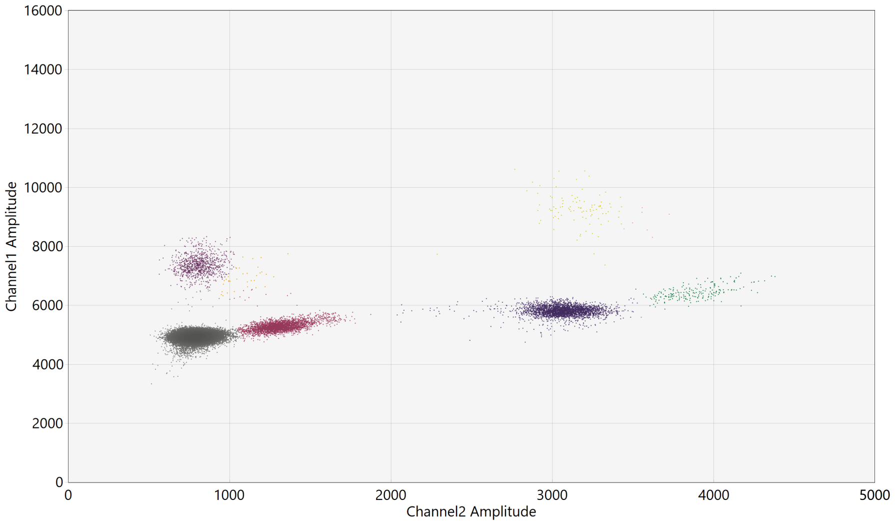

PP-5

FAM/HEX high: *HRAS* c.351G>C
FAM/HEX low: *TERT* C228T

Positive control

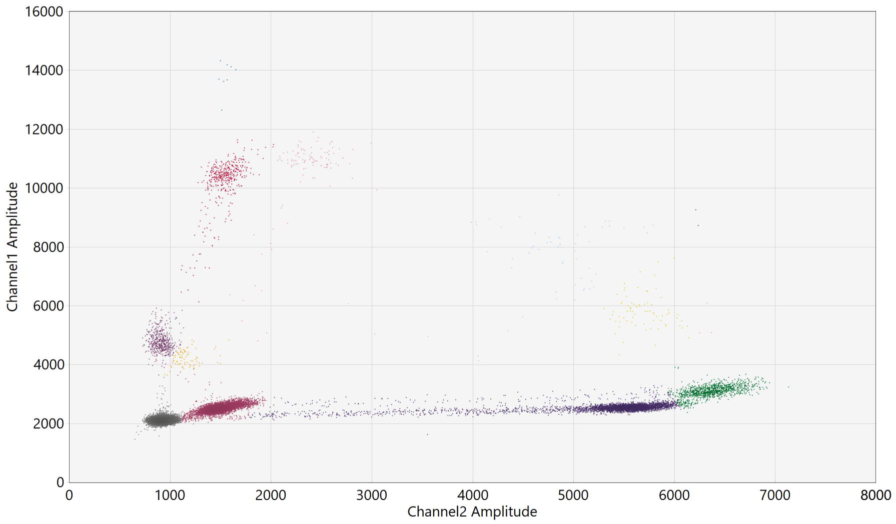

Negative control

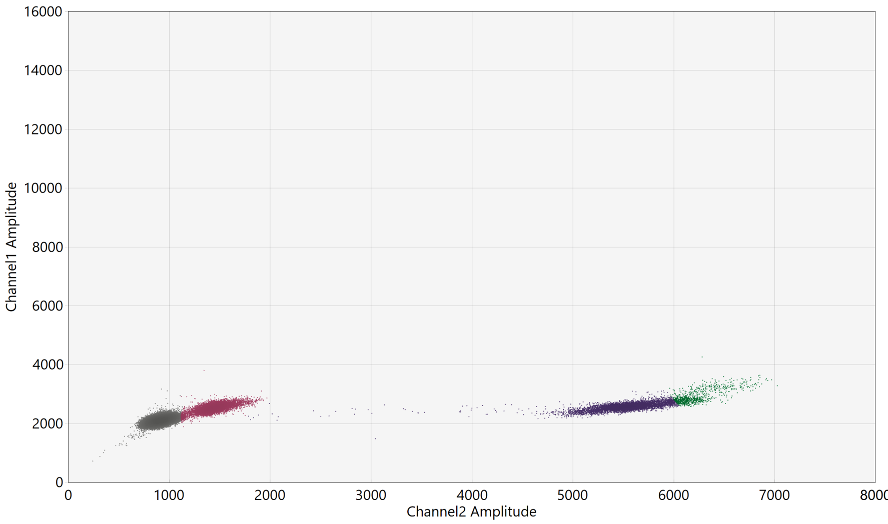

Plasma (follow-up)

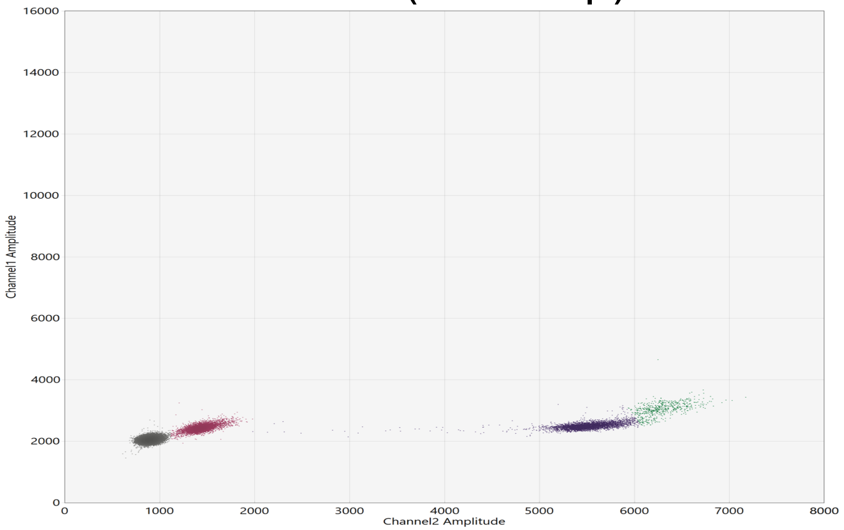

PP-6

FAM/HEX high: *PIK3CA* c.1633G>C
FAM/HEX low: *TP53* c.326T>C

Positive control

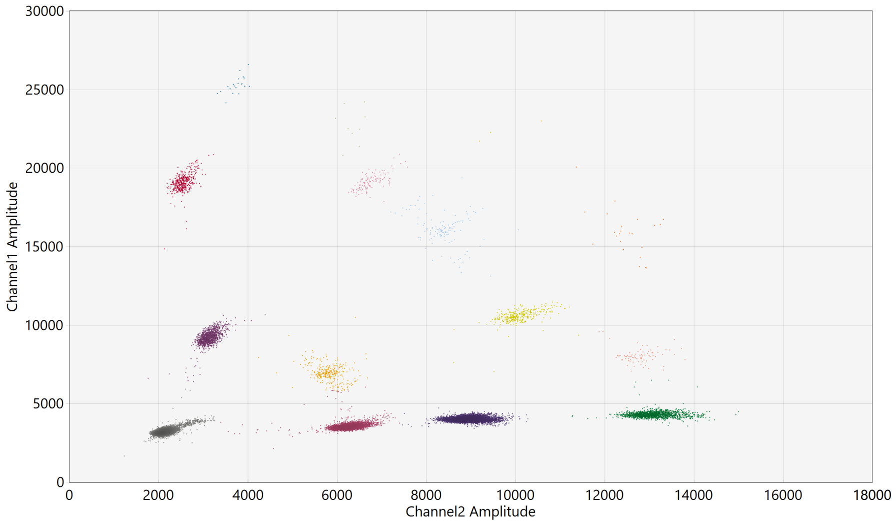

Negative control

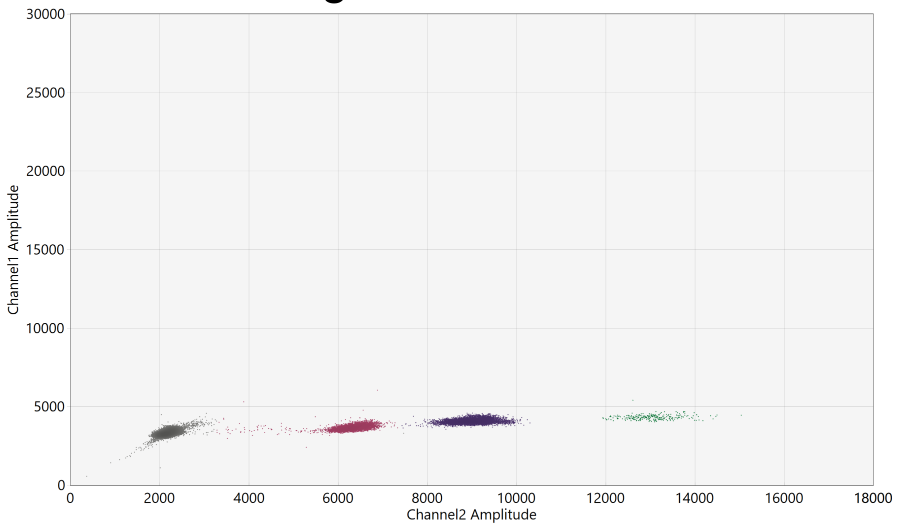

Plasma (diagnostic)

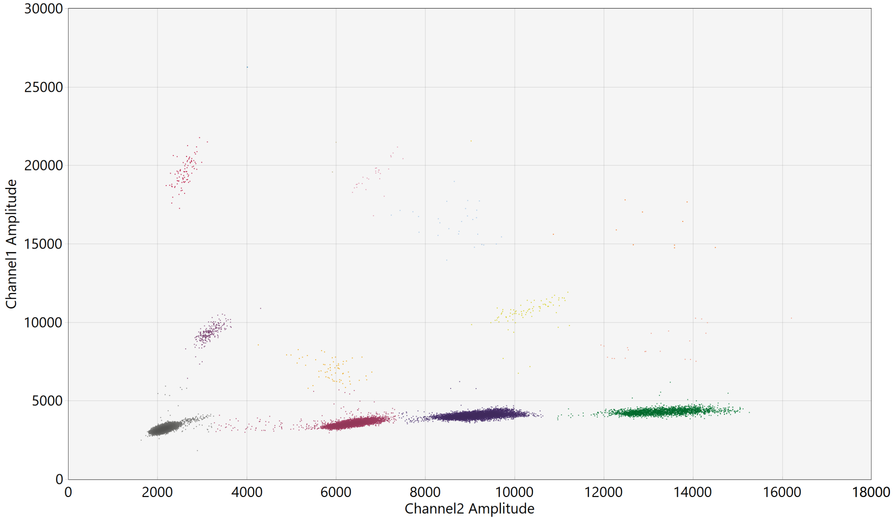

PP-7

FAM/HEX high: *KDM6A* c.3293delT
FAM/HEX low: *TERT* C228T

Positive control

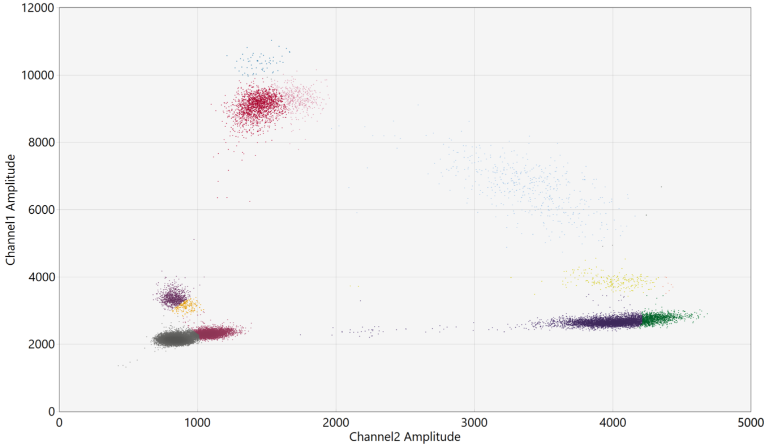

Negative control

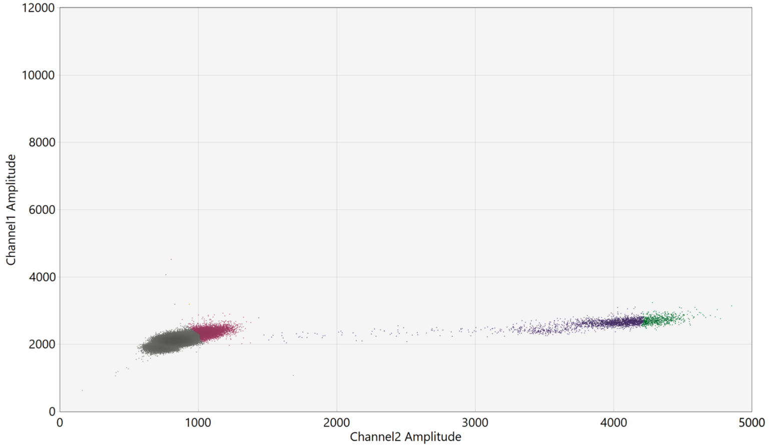

Plasma (diagnostic)

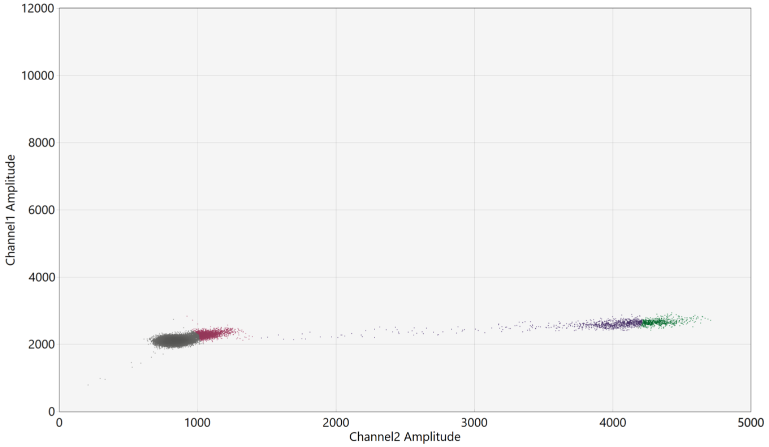

Plasma (follow-up)

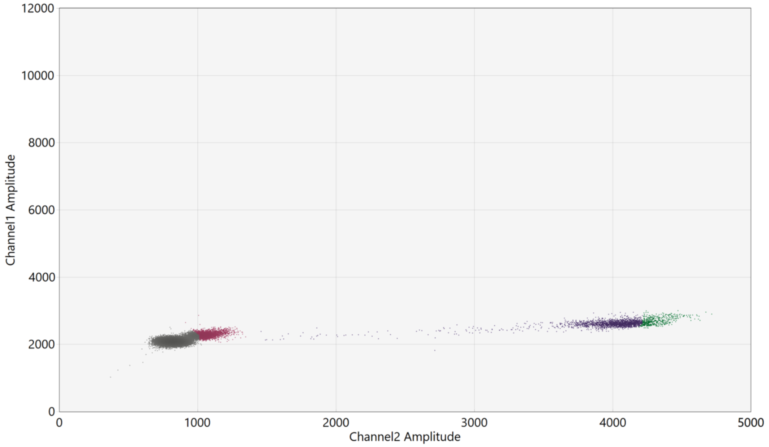

Plasma (follow-up)

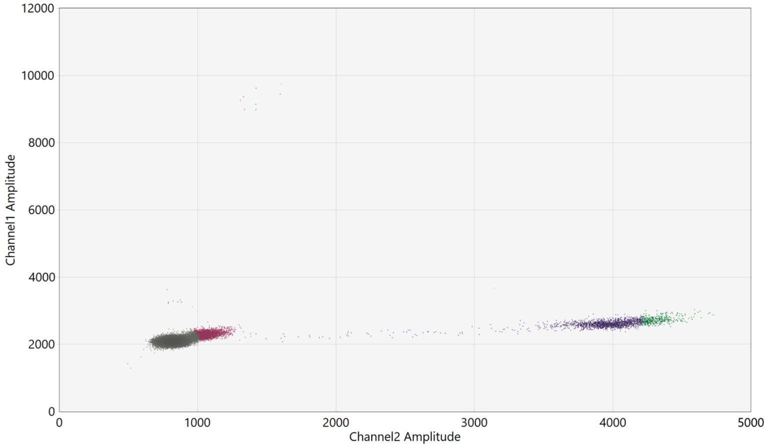

PP-8

FAM/HEX high: *TSC1* c.2507C>G
FAM/HEX low: *TERT* C228T

Positive control

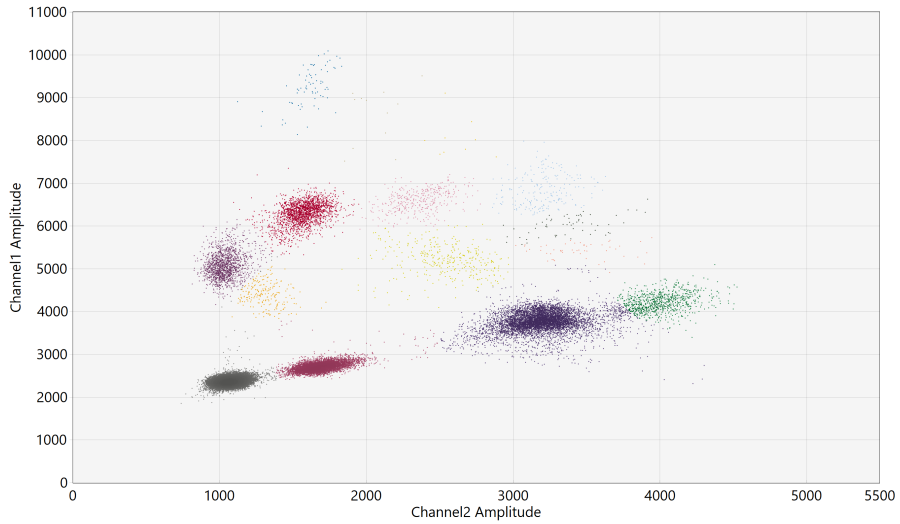

Negative control

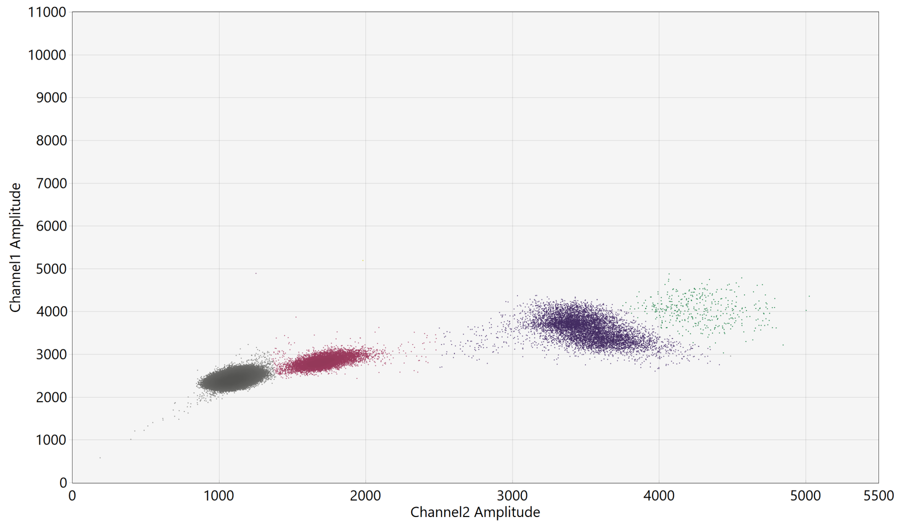

Plasma (diagnostic)

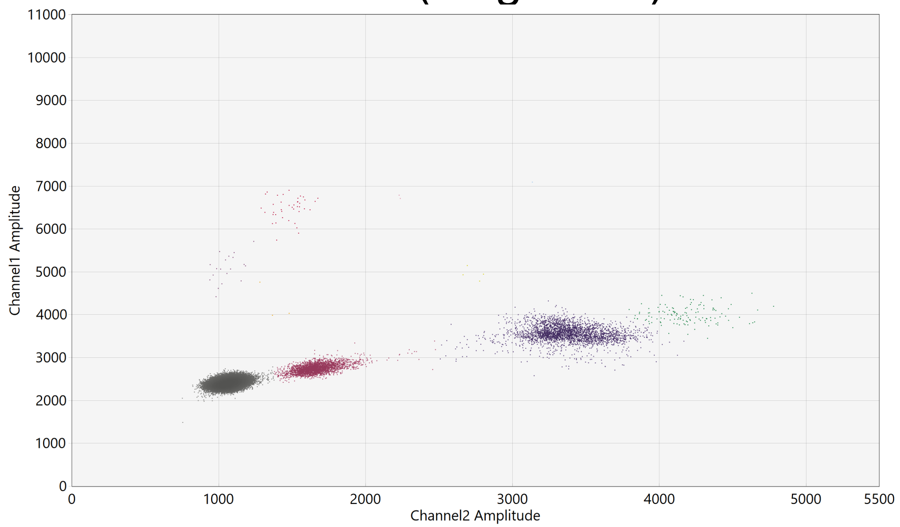

PP-9

FAM/HEX high: *KMT2D* c.11024delG
FAM/HEX low: *ELF3* c.740G>A

Positive control

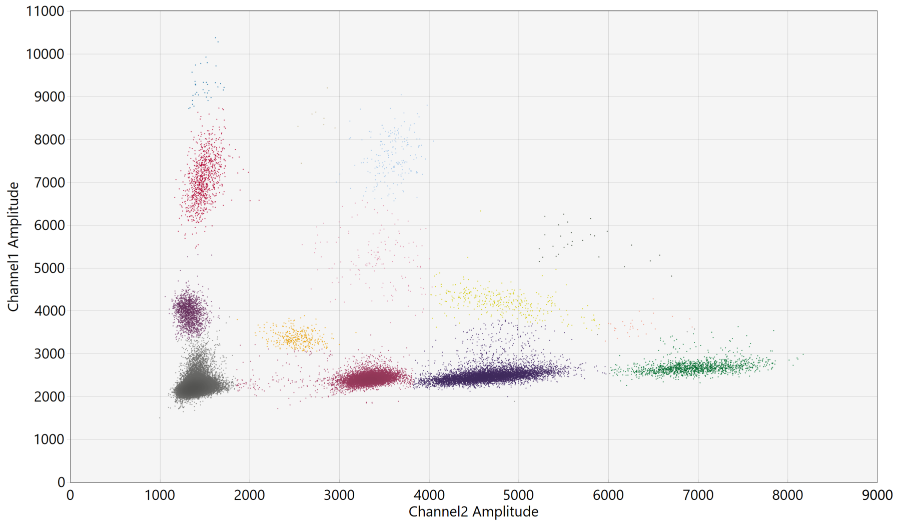

Negative control

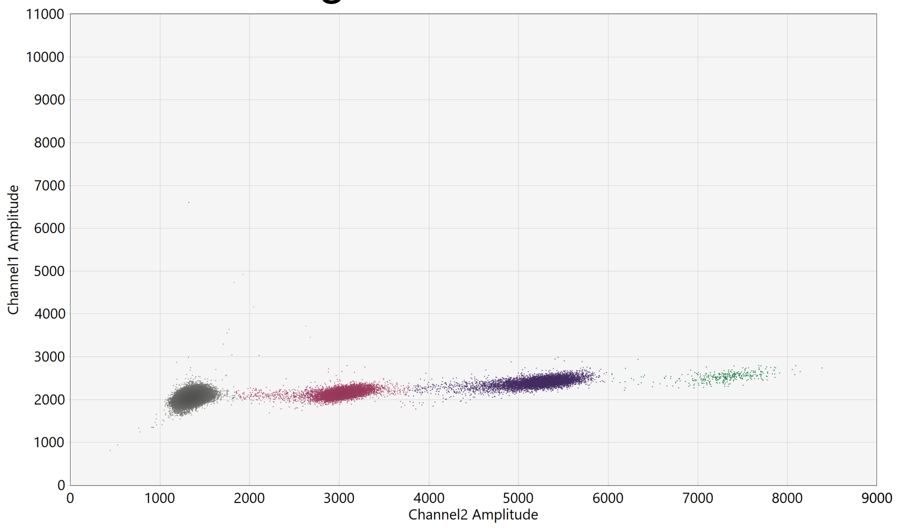

Plasma (diagnostic)

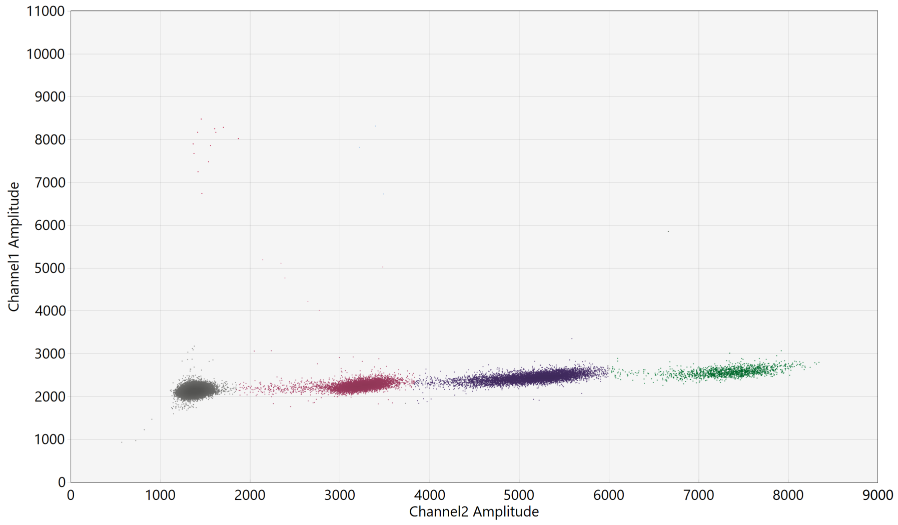

Supplement: Supplementary file 1 — Supplementary file1 (PDF 5674 KB) [file 345_2023_4583_MOESM1_ESM.pdf]
