# Supplementary material for: Patient-specific targeted analysis of circulating tumour DNA in plasma is feasible and may be a potential biomarker in UTUC
Source: World J Urol. 2023 Sep 18;41(12):3421–7. doi: 10.1007/s00345-023-04583-w (PMC10693512; doi:10.1007/s00345-023-04583-w)
Supplement: Supplementary file 2 — Supplementary file2 (PPTX 81 KB) [file 345_2023_4583_MOESM2_ESM.pptx]

## Slide 1
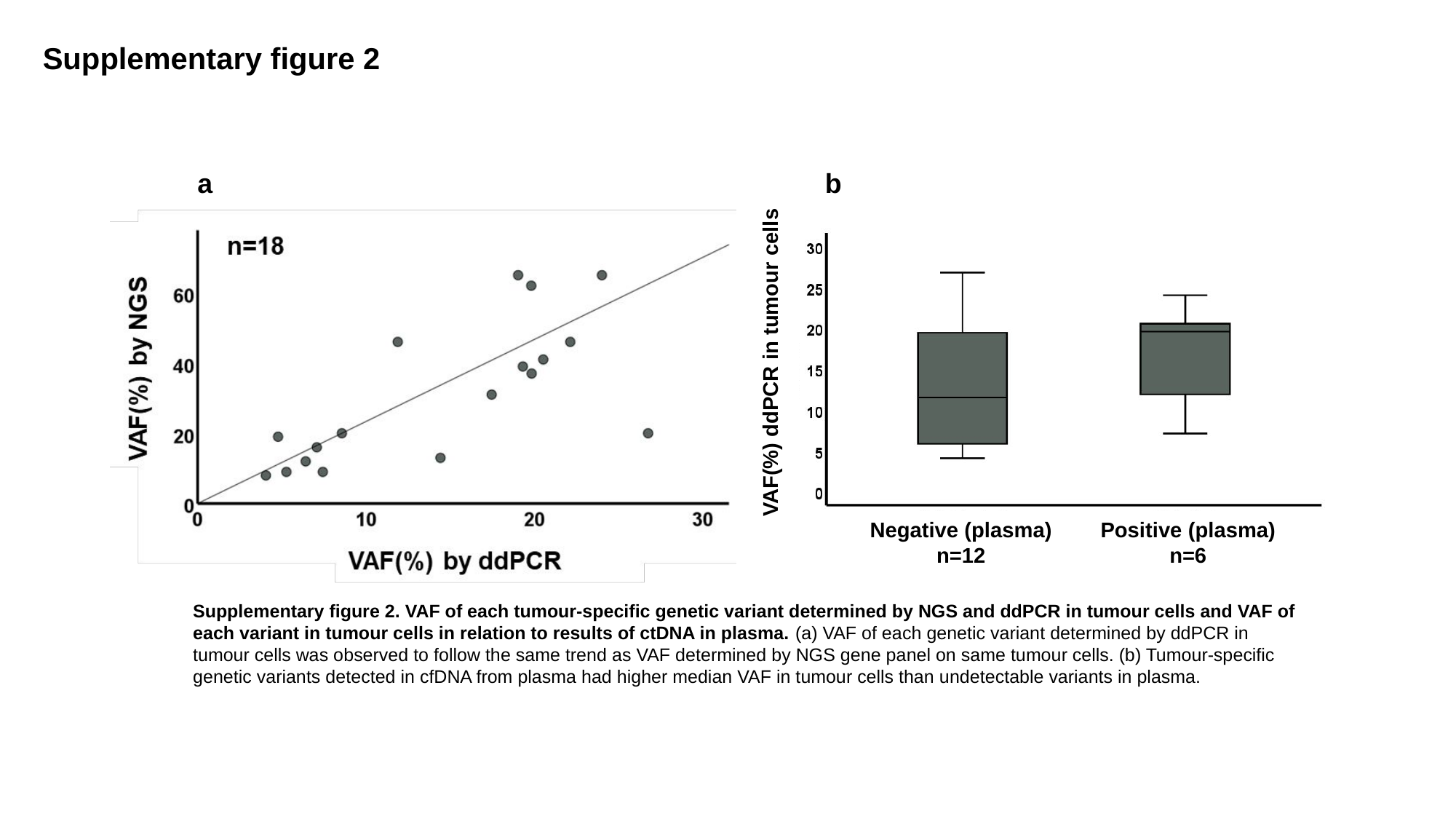

Supplementary figure 2
a
b
VAF(%) ddPCR in tumour cells
Negative (plasma)
n=12
Positive (plasma)
n=6
Supplementary figure 2. VAF of each tumour-specific genetic variant determined by NGS and ddPCR in tumour cells and VAF of each variant in tumour cells in relation to results of ctDNA in plasma. (a) VAF of each genetic variant determined by ddPCR in tumour cells was observed to follow the same trend as VAF determined by NGS gene panel on same tumour cells. (b) Tumour-specific genetic variants detected in cfDNA from plasma had higher median VAF in tumour cells than undetectable variants in plasma.
