# Supplementary material for: Patient-specific targeted analysis of circulating tumour DNA in plasma is feasible and may be a potential biomarker in UTUC
Source: World J Urol. 2023 Sep 18;41(12):3421–7. doi: 10.1007/s00345-023-04583-w (PMC10693512; doi:10.1007/s00345-023-04583-w)
Supplement: Supplementary file 3 — Supplementary file3 (DOCX 15 KB) [file 345_2023_4583_MOESM3_ESM.docx]

# Supplementary methods

UTUC patient cohort
Tumour grade was assessed by cytology or biopsy according to WHO classifications 1999 and 2004 for all included patients. Clinical data including age at diagnosis, sex, tumour size at URS and clinical outcome during follow-up was retrieved from a local database and medical records. Disease progression was defined as distant metastasis, progression to higher tumour grade or death from UTUC during follow-up and recurrence was defined as local recurrence in the upper urinary tract. All patients were followed until February 2023 or death.

Extraction of cfDNA from plasma
Plasma and cfDNA was extracted from blood samples collected in Streck® tubes (La Vista, NE, USA). In short, double centrifugation was carried out within 5 days from sampling to remove blood cells and plasma was stored in -80°C until use. cfDNA was extracted using QIAamp DNA Blood Mini Kit (Qiagen, Hilden, Germany) from 3-5mL plasma and eluted in 40µl AVE buffer.

Droplet Digital PCR and data analysis
Digital droplet PCR (ddPCR) was performed as previously published [1]. Targets for each patient were selected based on previous results from NGS gene panel where single nucleotide variants (SNV) or indels with highest variant allele frequencies (VAF) were primarily chosen. ddPCR mutation assays for each target labelled with fluorophores FAM for mutant allele and HEX for wildtype (wt) allele were purchased from Bio-Rad (Hercules, CA, USA). All used assays are listed in Supplementary Table 1 with their specific final annealing temperature. Amplitude multiplex ddPCR for two targets/patient was performed with the QX200 AutoDG Droplet Digital PCR System (Bio-Rad, Hercules, CA, USA) according to the manufacturer’s protocol on both cfDNA from plasma and tumour cell DNA. All reactions were run in a 22µl reaction volume as previously described and betaine was added when the multiplex assay contained the *TERT* C228T_88 probe. PCR amplifications were performed in SimpliAmp™ Thermal Cycler (Thermo Fisher, Waltham, MA, USA) with the following program: 95°C for 10 min, followed by 40 cycles of 94°C for 30 s and 55-60°C annealing temperature for 60 s and finally 98°C for 10 min and infinite hold at 4°C. All samples were run in triplicates. Tumour cell DNA was used as positive control while normal cfDNA from healthy blood donors was used as negative control. ddPCR data analysis was then performed using QuantaSoft Analysis Pro software (Bio-Rad, Hercules, CA, USA) according to manufacturer’s guidelines. Thresholds for discrimination between positive and negative droplets was performed by visualising amplitude plots for positive and negative controls. VAF and concentration of mutant and wild type allele in plasma (copies/mL) was then calculated based on merged concentration (copies/well) output. The false positive rate was calculated as VAF of mutant allele in negative control.

Reference:

1. Wallander K, Haider Z, Jeggari A, Foroughi-Asl H, Gellerbring A, Lyander A, Chozhan A, Cuba Gyllensten O, Hägglund M, Wirta V et al. (2023) Sensitive Detection of Cell-Free Tumour DNA Using Optimised Targeted Sequencing Can Predict Prognosis in Gastro-Oesophageal Cancer. Cancers (Basel) 15
