# Supplementary material for: Patient-specific targeted analysis of circulating tumour DNA in plasma is feasible and may be a potential biomarker in UTUC
Source: World J Urol. 2023 Sep 18;41(12):3421–7. doi: 10.1007/s00345-023-04583-w (PMC10693512; doi:10.1007/s00345-023-04583-w)
Supplement: Supplementary file 4 — Supplementary file4 (DOCX 14 KB) [file 345_2023_4583_MOESM4_ESM.docx]

| **Supplementary table 1. List of all ddPCR assays used in the study.** | | | | | |
| --- | --- | --- | --- | --- | --- |
| **ID** | **Target** | **Assay ID** | **Company** | **Annealing temp** | **Comment** |
| PP-2, PP-4, PP-5, PP-7, PP-8 | TERT C228T_88 | dHsaEXD20945488 | Bio-Rad | 60/57 | Betaine |
| PP-1 | KMT2D c.15517G>T | dHsaMDM3128068901 | Bio-Rad | 55 |  |
| PP-1 | KMT2D WT for c.15517G>T | dHsaMDW3128068903 | Bio-Rad | 55 |  |
| PP-1 | KMT2D c.6562delG | dHsaMDM5073289971 | Bio-Rad | 55 |  |
| PP-1 | KMT2D WT for c.6562delG | dHsaMDW5073289973 | Bio-Rad | 55 |  |
| PP-2 | KDM6A p.Q555* c.1663C>T | dHsaMDM1912718951 | Bio-Rad | 60 | Betaine |
| PP-2 | KDM6A WT for p.Q555* c.1663C>T | dHsaMDW1912718953 | Bio-Rad | 60 | Betaine |
| PP-3 | KDM6A c.3373dupA | dHsaMDM7298952081 | Bio-Rad | 55 |  |
| PP-3 | KDM6A WT for c.3373dupA | dHsaMDW7298952083 | Bio-Rad | 55 |  |
| PP-3 | FGFR3 p.Y373C | dHsaMDM8876711461 | Bio-Rad | 55 |  |
| PP-3 | FGFR3 WT for p.Y373C | dHsaMDW8876711463 | Bio-Rad | 55 |  |
| PP-4 | KMT2C p.Q1478* | dHsaMDM1221763781 | Bio-Rad | 57 | Betaine |
| PP-4 | KMT2C WT for p.Q1478* | dHsaMDW1221763783 | Bio-Rad | 57 | Betaine |
| PP-5 | HRAS p.K117N c.351G>C | dHsaMDM5273612661 | Bio-Rad | 57 | Betaine |
| PP-5 | HRAS WT for p.K117N c.351G>C | dHsaMDW5273612663 | Bio-Rad | 57 | Betaine |
| PP-6 | TP53 p.F109S | dHsaMDM6375174891 | Bio-Rad | 55 |  |
| PP-6 | TP53 WT for p.F109S | dHsaMDW6375174893 | Bio-Rad | 55 |  |
| PP-6 | PIK3CA p.E545Q | dHsaMDM2559144441 | Bio-Rad | 55 |  |
| PP-6 | PIK3CA WT for p.E545Q | dHsaMDW2559144443 | Bio-Rad | 55 |  |
| PP-7 | KDM6A c.3293delT | dHsaMDM7909565181 | Bio-Rad | 60 | Betaine |
| PP-7 | KDM6A WT for c.3293delT | dHsaMDW7909565183 | Bio-Rad | 60 | Betaine |
| PP-8 | TSC1 p.S836* | dHsaMDM4761265711 | Bio-Rad | 60 | Betaine |
| PP-8 | TSC1 WT for p.S836* | dHsaMDW4761265713 | Bio-Rad | 60 | Betaine |
| PP-9 | ELF3 p.R247Q c.740G>A | dHsaMDM2385673251 | Bio-Rad | 55 |  |
| PP-9 | ELF3 WT for p.R247Q c.740G>A | dHsaMDW2385673253 | Bio-Rad | 55 |  |
| PP-9 | KMT2D c.11024delG | dHsaMDM8751826091 | Bio-Rad | 55 |  |
| PP-9 | KMT2D WT for c.11024delG | dHsaMDW8751826093 | Bio-Rad | 55 |  |
